# Uretero-Sciatic Hernia (Lindbom Hernia): A Case Report and Long-Term Follow-Up

**DOI:** 10.7759/cureus.31211

**Published:** 2022-11-07

**Authors:** Mohamed Mustafa, Lokesh Suraparaju, Marc Lyons, Suresh Gupta

**Affiliations:** 1 Urology, James Paget University Hospitals NHS Foundation Trust, Gorleston-on-Sea, GBR

**Keywords:** sciatic ureteral hernia, curlicue ureter sign, ureteral hernia, lindbom hernia, uretero-sciatic hernia

## Abstract

Ureteric herniation through the posterior pelvic wall is one of the rarest variants of hernias and causes of ureteric obstruction. The clinical features span from asymptomatic to a presentation with severe flank pain and life-threatening infection secondary to ureteric obstruction. The diagnosis needs a high index of suspicion and timely, appropriate radiological investigation. This article presents a case report of a patient who presented with a history of nonspecific abdominal pain and was diagnosed with a left-sided uretero-sciatic hernia (Lindblom hernia). This was managed with routine ureteral stent changes. Long-term follow-up and results from over 10 years of management are presented.

## Introduction

A ureteric herniation is generally a rare event, with the majority of cases being part of inguinoscrotal hernias. Historically, cases were incidentally reported intraoperatively during surgeries for inguinal hernias repair [1]. Herniation of the ureter through the sciatic foramina is extremely rare compared to inguinoscrotal herniation of ureters with less than 40 cases reported [2]. The first case was reported by Lindbom in 1947 [3]. In this article, we present a case report of uretero-sciatic hernia with a highlight on diagnosis and long-term management.

## Case presentation

A 52-year-old female presented with recurrent episodes of left loin pain over a period of 6 months in 2011. Her renal function tests were normal, however, the urine dipstick test was positive for hematuria. She has a duplex ureter on the left side and pyeloplasty of the right renal pelvis in 1970 apart from her medical treatment for essential hypertension.

**Figure 1:**
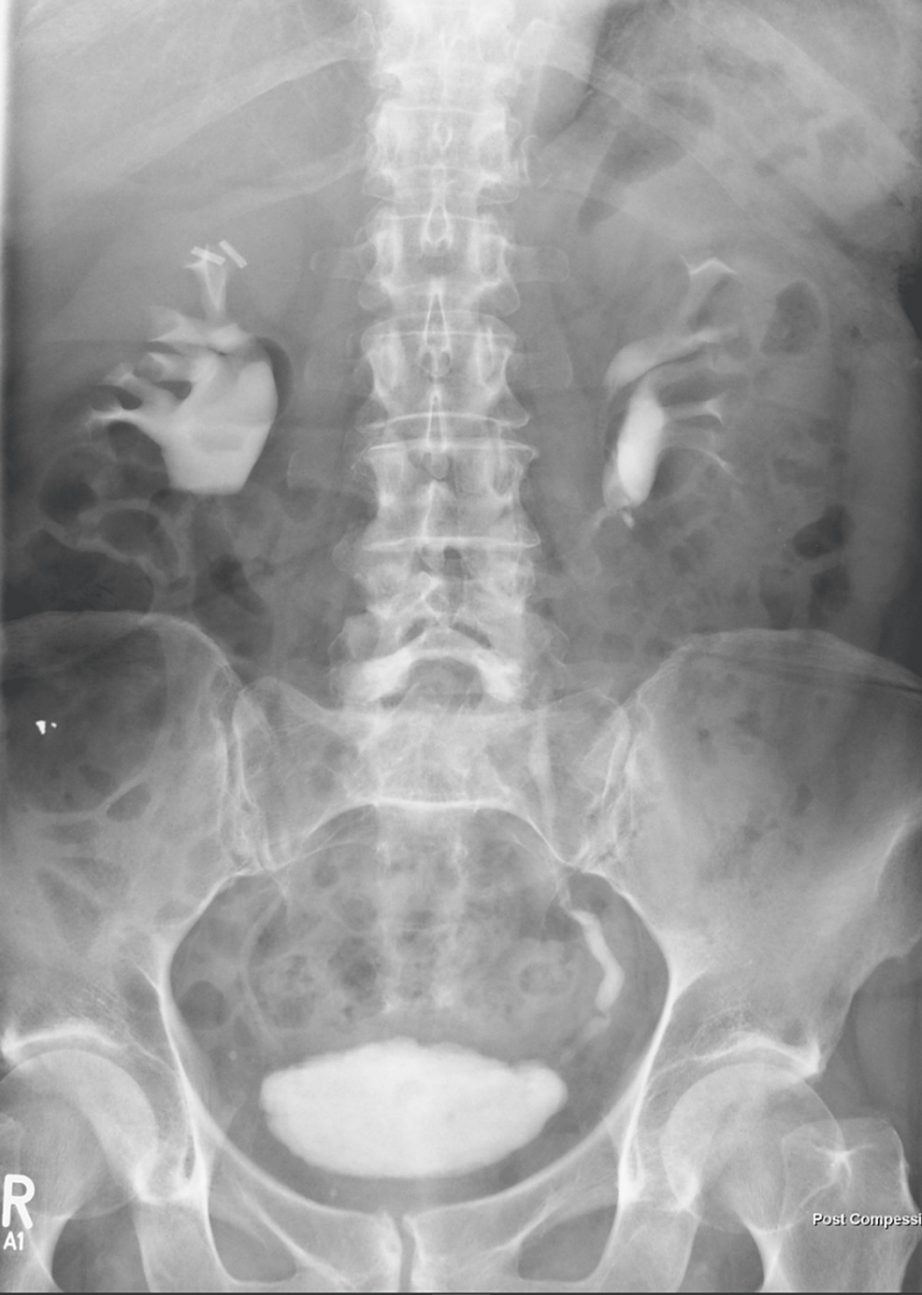
Intravenous Urogram (IVU) showing normal ureteric contrast flow with mild fullness of the right renal pelvis.

Initially, her presentation was addressed as a ureteric colic for possible urological stone disease. However, all her initial investigations with Ultrasound and Computed Tomography (CT) scans of the urinary system came back with no significant abnormalities apart from mild dilatation of the left pelvicalyceal system which did not explain her symptomatology. With the pain being recurrent, an intravenous Urogram (IVU) (Figure 1) was done which did not show any obvious signs of obstructive uropathy. At that time the patient was reassured and discharged with no further follow-up.

**Figure 2:**
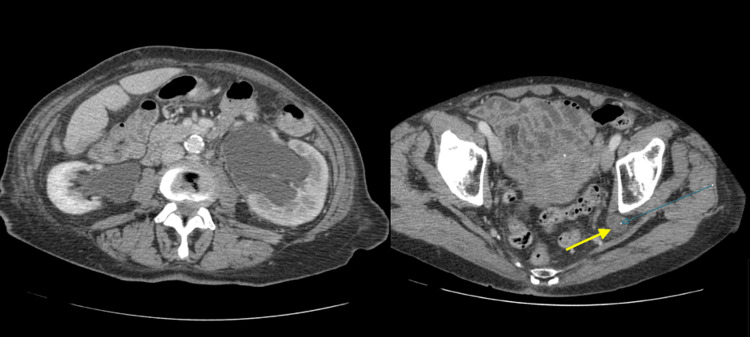
CT scan showing severe hydronephrosis on the left side with herniation of the left ureter through the great sciatic foramen (arrowed).

Two years later, the patient presented with persistent severe left flank pain associated with a decline in renal function and raised inflammatory markers. A CT scan showed severe left-sided hydro-nephrosis, and the diagnosis of Lindbom hernia (Figure 2) was made. The patient then was admitted to the hospital and had a left-sided nephrostomy tube insertion, which relieved the obstruction and improved the renal function back to normal baseline.

**Figure 3:**
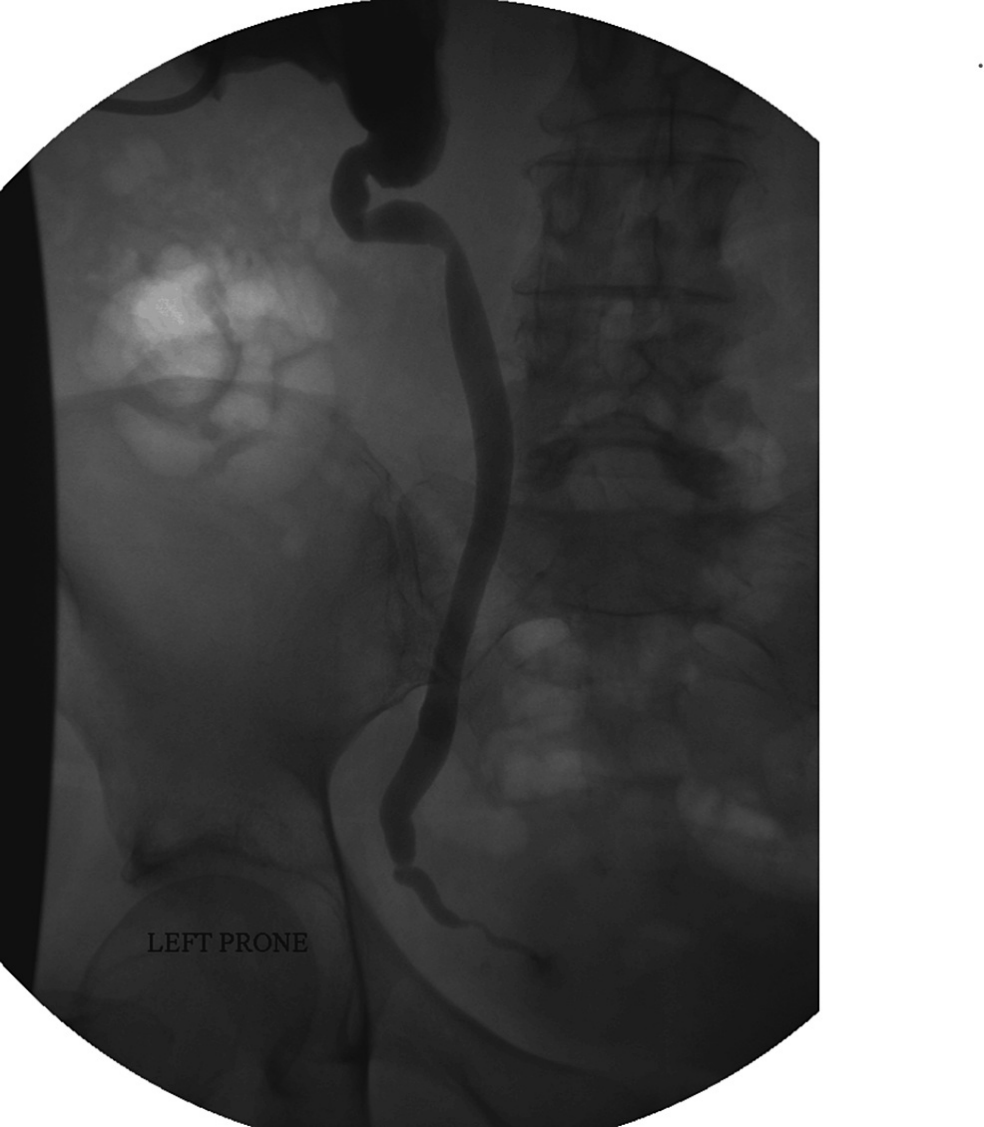
Contrast flew on the affected side with the resolution of the hernia.

The patient came back a few weeks later for the insertion of an ante-grade stent. However, the nephrostogram showed that the hernia has reduced (Figure 3). In light of this, the stent was not inserted, and the nephrostomy tube was removed.

**Figure 4:**
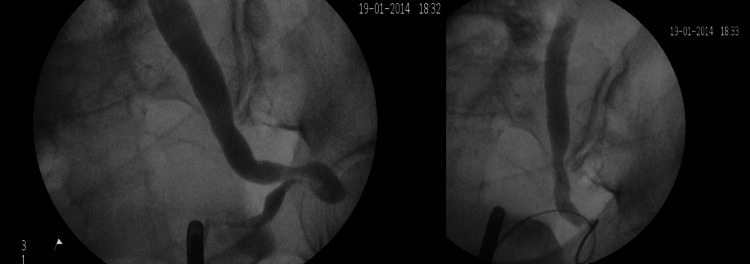
Attempted retrograde stenting.

A couple of months later, the patient presented again, unwell and in severe pain. A repeat CT scan showed a recurrence of the hernia. The patient was then planned to go for an emergency retro-grade stenting which unfortunately failed due to the severe kinking of the distal ureter into the sciatic foramen (Figure 4).

**Figure 5:**
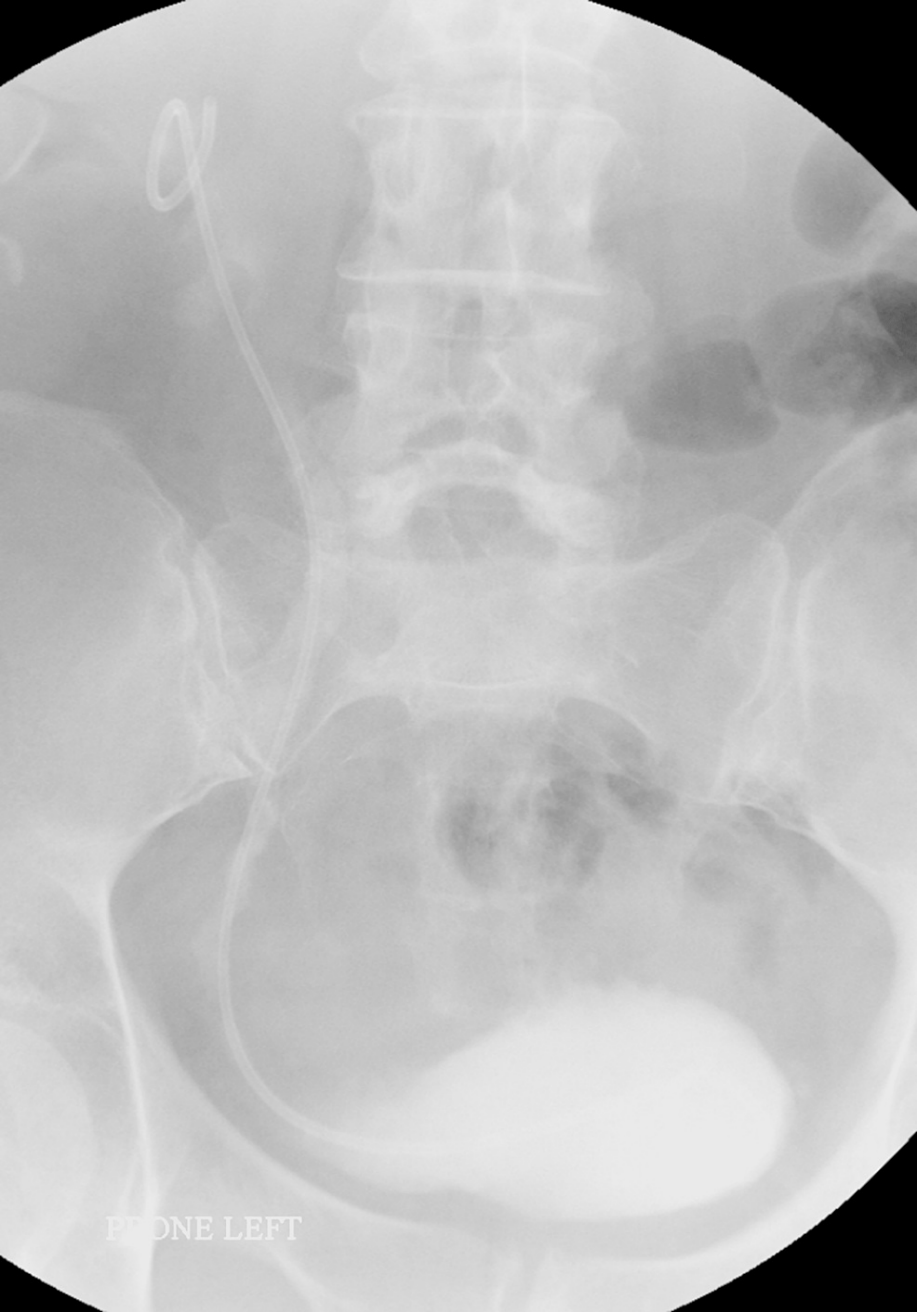
Antegrade stenting with reduction of the hernia.

The patient then had an emergency nephrostomy followed by antegrade stenting, which reduced the hernia (Figure 5).

The patient recovered very well afterward. A re-implantation of the ureter was offered as a permanent solution to the patient's problem; however, she was not particularly keen on extensive surgery, whether laparoscopic or open surgery. Since 2014, in her 80s now, the patient has been managed with six monthly retrograde changes of ureteric stents.

## Discussion

Uretero-sciatic hernias are rare disease entities with no more than 40 cases published worldwide including this case. Uretero-sciatic hernias are thought to be secondary to a partial loss of pelvic fascia, atrophy of the piriformis, adhesions, congenital deformity, or a combination of either [4]. The ureters commonly herniate in the supra-piriformis compartment of the greater sciatic foramen [5].

The clinical presentation of uretero-sciatic hernia varies and can range from asymptomatic to life-threatening urinary sepsis or obstructive uropathy. Patients may complain of flank or abdominal pain which can be intermittent and colicky [6], as was the first presentation in our case.

Since the clinical features of uretero-sciatic hernias are rather non-specific, the diagnosis is usually dependent on the radiographic investigation which might include intravenous urogram, retrograde pyelography, or computed tomography. The Curlicue sign is described as ureteric obstruction with U-shaped tortuosity through the sciatic foramen which is pathognomonic of uretero-sciatic hernia [7]. Interestingly, in a recently reported case similar to this case, an initial CT scan did not show any signs or evidence of sciatic herniation. However, when the patient presented with severe urological-sepsis, a repeated CT scan was diagnostic for an obstructed uretero-sciatic hernia. This anatomical fact implies that these hernias are reducible in nature and hence, might be asymptomatic, or only present as intermittent loin pain. This feature is of clinical significance as it means that at the time of imaging, it might be inconclusive [8]. This condition can re-occur and hence, permanent long-term management should be planned [9]. Early recognition and treatment are key, where recovery of normal renal function is proved by radioisotope images like MAG3 indicating reversible obstructive uropathy [9].

Generally, management options include observation (mostly for asymptomatic patients), ureteral stenting, and surgical correction. As septic and acutely unwell patients are at substantial risk for general anesthesia and surgical procedures, decompressing the system using nephrostomies has been reported [9]. Most of the reported cases have been either initially or definitely managed with ureteric stenting, both via retrograde or antegrade approaches [4]. However, the condition can be very severe where stenting does not relieve the obstructing ureteric deformity and maintain drainage of the system [10]. In similar cases, open, laparoscopic, and robotic approaches have been described for the reduction of ureter and hernia repair.

## Conclusions

Our case report highlights the challenges in the diagnosis and management of this rare condition. However, after around a decade of follow-up, it also proves that stenting alone can be the main course of management without the need for aggressive surgical treatment unless in circumstances where stenting fails to reverse the condition. As it is a rare case, bringing awareness of the significant symptomatology will necessitate or stimulate our thinking about such rare causes where no obvious cause can be identified.
